# Long-Term Evaluation of Pulp Vitality Preservation in Direct and Indirect Pulp Capping: A Retrospective Clinical Study

**DOI:** 10.3390/jcm13133962

**Published:** 2024-07-06

**Authors:** Mario Alovisi, Andrea Baldi, Allegra Comba, Roberta Gamerro, Gaetano Paolone, Mauro Mandurino, Mario Dioguardi, Andrea Roggia, Nicola Scotti

**Affiliations:** 1 Department of Surgical Sciences, Dental School, University of Turin, 10124 Turin, Italy; andrea.baldi@unito.it (A.B.); allegra.comba@unito.it (A.C.); roberta.gamerro@gmail.com (R.G.); zoltan89@msn.com (A.R.); nicola.scotti@unito.it (N.S.); 2 Department of Dentistry, Dental School, IRCCS San Raffaele Hospital, Vita-Salute University, 20123 Milan, Italy; paolone.gaetano@hsr.it (G.P.); m.mandurino@studenti.unisr.it (M.M.); 3 Department of Clinical and Experimental Medicine, University of Foggia, 71122 Foggia, Italy; mario.dioguardi@unifg.it

**Keywords:** direct pulp capping, indirect pulp capping, self-etch adhesive, mineral trioxide aggregate, resin-based glass ionomer, pulp vitality

## Abstract

**Background:** This retrospective clinical study aimed to assess dental pulp tissue reactions to direct and indirect pulp capping after 10 years of follow-up. **Methods:** A total of 276 permanent teeth with deep carious lesions were evaluated and divided into five groups: Group (1), direct pulp capping with Mineral Trioxide Aggregate cement; Group (2), direct pulp capping with a resin-based glass ionomer; Group (3), direct pulp capping with TheraCal; Group (4), indirect pulp capping with a three-step total-etch adhesive system; and Group (5), indirect pulp capping with a two-step self-etch adhesive system. **Results:** A 72.5% success rate was achieved overall. A statistically significant difference was found when comparing direct and indirect pulp capping with a success rate of 23.8% and 93.8%, respectively. For direct pulp-capping procedures, the area of pulp exposure was correlated with pulp necrosis (*p* = 0.035), while bleeding after exposure appeared independent (*p* = 0.053). Patient age was significantly related to the maintenance of pulp vitality (*p* = 0.013). A statistically significant correlation between the pulp-capping material and the occurrence of pulp necrosis was discovered (*p* = 0.017). For the indirect pulp-capping treatments, a significant correlation between patient age (*p* = 0.021) and the adhesive system (*p* = 0.019) with pulp necrosis was described. **Conclusions:** The pulp-capping material, patient age, and the width of the pulp exposure before the procedure should be carefully considered when performing direct pulp-capping treatments. The performance of the etch-and-rinse adhesive systems was superior to the self-etch system during the indirect pulp-capping procedures.

## 1. Introduction

When a tooth is subjected to trauma, carious lesions, or restorative procedures, dental pulp vitality preservation overtime is one of the major concerns for the clinician [1,2]. In the case of deep cavities that penetrate both enamel and dentin, indirect or direct pulp-capping procedures could be required depending on the lesion depth and its proximity to pulp tissue after caries debridement [3]. Direct pulp capping consists of applying biocompatible materials directly to the exposed pulp where the odontoblast layers have been breached, while indirect pulp capping leaves a thin dentin layer [3]. The main goals of any pulp-capping procedure are to control bacterial infection by stopping the progression of any residual caries, to encourage the formation of new tertiary dentin, and to provide a biocompatible and long-lasting seal that shields the pulp complex from noxious agents and bacteria [4]. Hence, a variety of pulp-capping materials have been suggested overtime that could be settled in proximity to the pulp tissue to promote reactionary or reparative dentin formation. 

Direct pulp capping seeks to improve reparative dentin formation and odontoblast-like cell differentiation from dental pulp stem cells [3]. For many years, calcium hydroxide (Ca(OH)2) has been widely used for direct pulp capping [5]. Afterwards, the Mineral Trioxide Aggregate (MTA), a bioactive and biocompatible cement, was introduced [6]. According to several studies published in the literature, the MTA appeared more efficient than Ca(OH)2 because it caused less inflammation and more uniform hard tissue/dentin bridge formation [6,7,8,9]. Moreover, glass ionomer sealers, even in conjunction with a resin composite, have been proposed for pulp capping [10]. A persistent inflammatory response mediated by macrophages and giant cells and a large zone of pulp necrosis are caused by the diffusion of these materials into the pulp tissue [10,11]. However, the extensive zone of necrosis around the glass ionomer components can be replaced by fibroblast proliferation and fibro-dentine deposition, creating a calcific barrier [10,11]. Nevertheless, despite the controversy surrounding its use for pulp capping, the high fluoride release makes this material suitable for restorations, especially in pediatric patients [10,12]. TheraCal (Bisco Dental, Richmond, Canada), a resin-modified calcium silicate-filled material, was also introduced for pulp capping. According to recent studies, TheraCal seemed to release significantly more calcium than the MTA and calcium hydroxide, encouraging the formation of apatite and the differentiation of the odontoblasts that lead to the production of new dentin [6,13]. Additionally, the release of calcium is essential for osteopontin and BMP-2 modulation during pulp calcification as well as for the proliferation of odontoblast-like cells [14]. Thus, this light-curable capping material with the ability to polymerize to a considerable depth may avoid the risk of untimely dissolution and could be able to favor apatite formation, inducing odontoblast differentiation [6].

When considering indirect pulp capping, the application of adhesive systems as indirect pulp-capping materials within the composite restoration is currently recommended [15]. Deep carious lesions are usually sealed with adhesive systems with or without cavity liners, being the protection from bacterial microleakage mandatory for pulp vitality preservation [15,16,17]. However, the pulp may indeed be harmed by several factors, including the etching agent, the toxicity of the leached components, the microleakage due to polymerization shrinkage, the nano-leakage and sensitization caused by the partial bonding penetration into the demineralized dentin, and the temperature rise during setting [18]. Etch-and-rinse systems employ phosphoric acid to etch enamel and dentin prior to the application of the bonding solution, and the smear layer is removed, increasing the dentin permeability [19]. Over-etching or incomplete monomer application may lead to the creation of voids in the hybrid layer as well as the presence of denude collagen fibrils and nano-leakage [20]. Thus, self-etch systems have been introduced, as they do not remove but incorporate the smear layer into the hybridized complex with also the advantage of being less technique-sensitive [21]. However, the perception that self-etch adhesives may cause less nano-leakage and post-operative sensitivity in deep cavities is not fully supported by evidence-based findings [19,20,21]. 

This retrospective clinical study examined the long-term outcomes of direct and indirect pulp capping performed with different materials after ten years of follow-up. The primary null hypothesis is that the type of pulp capping used, the intra-operative findings, and patient age have no effects on the long-term results of the direct pulp-capping procedures. The second null hypothesis is that the adhesive system does not affect the pulp vitality maintenance after indirect pulp capping over time.

## 2. Materials and Methods

This study was approved by the local ethical committee (Protocol Number 06CS1123), and the protocol followed the recommendations of the Declaration of Helsinki for investigations with human subjects [22].

### 2.1. Study Characteristics, Participants, and Design

For this retrospective clinical evaluation, the data of the patients treated with direct or indirect pulp capping between May 2009 and July 2013 with at least 10 years of follow-up were analyzed. All patients were divided into 5 groups according to the treatment, the material, and the adhesive system used: Group (1), direct pulp capping with Mineral Trioxide Aggregate (MTA) cement; Group (2), direct pulp capping with a resin-based glass ionomer; Group (3), direct pulp capping with TheraCal (Bisco Dental, Richmond, BC, Canada); Group (4), indirect pulp capping with a 3-step total-etch adhesive system; Group (5), indirect pulp capping with a 2-step self-etch adhesive system. 

### 2.2. Enrollment and Inclusion Criteria

Patients presenting a diagnosis of a permanent tooth with deep primary carious lesions and pulp exposure or a thin affected dentin layer between the pulp and the cleaned cavity were considered in this study. The inclusion criteria were the absence of pulp symptoms, spontaneous or percussion pain, or periodontal pathology. Patients without medical comorbidities or pharmaceutical treatment that may influence the treatment outcomes and the follow-up period were considered for the study after the revision of a complete medical and dental anamnestic questionnaire. The data about pulp vitality tests (thermic and electric), periapical radiography, periodontal probing, and percussion tests were reviewed as diagnostic procedures. The following conditions were considered as exclusion criteria: irreversible pulpitis or pulp necrosis, history of unprovoked toothaches, periodontal inflammation, grade II-III mobility, enamel and dentinal cracks, furcation/apical radiolucency, and radiographic evidence of internal/external resorption or pulp calcification. Moreover, teeth requiring indirect restorations were excluded from the study. 

### 2.3. Clinical Procedures

All treatments were performed by a single experienced operator following a standardized protocol. After local anesthesia with 1:100.000 Mepivacaine and isolation with a rubber dam (NicTone, MDC Dental, Ciudad de Mexico, Mexico), the carious lesion was removed under 3.5× magnification. High-speed round-shaped diamond burs (#801.001.014, Komet Dental, Lemgo, Germany) mounted on a high-speed handpiece with water and air cooling were employed for enamel removal. Round, steel slow-speed burs (#4S.001.014, Komet Dental, Lemgo, Germany) with water and air spray coolant and sharp hand excavators (EXCE2 and EXCE3; Hu-Friedy, Chicago, IL, USA) were used for caries removal. Caries removal continued even after pulpal exposures until dentin resisted hand excavation with a sharp excavator as per final inspection. Finally, enamel margins were finished with fine-grit burs. Before the direct pulp-capping procedure, intra-operative information was collected among the patient’s clinical data, such as the bleeding level of the exposed pulp (absent, minimal, or moderate bleeding) and the width of the exposure area (less or about 1 mm). Moreover, detailed clinical images with a digital dedicated camera (Canon Eos 450D, Ōta, Tokyo, Japan—Optical Macro Sigma 105 mm. 2-8-15 Kurigi, Asao-ku, Kawasaki-shi, Kanagawa 215-0033 Japan) of the intra-operative procedures were kept as part of routine clinical practice. In the case of minimal pulp bleeding, hemostasis was controlled by rinsing with physiological saline-soaked pellets kept in place for 10–20 s. No other material was used so as to not create an interaction with the pulp-capping material selected for the study. Pulp bleeding for more than 10 min from pulp exposure was considered among the exclusion criteria. When the pulp exposure occurred with moderate bleeding, teeth were treated with direct pulp capping with different clinical strategies according to the dental material employed. All these clinical data and images were carefully described in the patients’ folders.

Group 1: Direct pulp capping with Mineral Trioxide Aggregate cement (MTA) (ProRoot MTA, Dentsply Sirona, Maillefer, Switzerland). The MTA was applied on the pulp wound and dried with a cotton pellet. Dyract flow (Dentsply Sirona, Maillefer, Switzerland) was set on the MTA and light-cured to avoid MTA removal during the subsequent adhesive procedures. Then, a 2-step self-etch adhesive system was employed (Clearfil SE Bond, Kuraray, Tokyo, Japan): it was placed with a micro-brush in two layers, and light-curing for 20 s with an LED curing light at 1000 mW/cm^2^ was performed (Starlight, Mectron, Carasco, Italy). 

Group 2: Direct pulp capping with a 1 mm layer of resin-based glass ionomer (Fuji IX, GC, Aichi, Japan). Once setting was completed, the 2-step self-etch adhesive system was applied as described in Group 1. 

Group 3: Direct pulp capping with a 1 mm layer of TheraCal (Bisco Dental, Richmond, BC, Canada) was applied on the pulp exposure and light-cured for 20 s with an LED curing light at 1000 mW/cm^2^ (Starlight, Mectron, Carasco, Italy). Subsequently, the 2-step self-etch adhesive system was applied as described in Group 1. 

When the pulp exposure did not occur and a thin affected dentin layer of approximately 1 mm remained between the pulp and the cleaned cavity, the teeth were treated with indirect pulp capping, randomly assigned to different groups according to the adhesive system employed, which was applied according to the manufacturer’s instructions. All these clinical data and images were carefully described in the patients’ folders.

Group 4: Indirect pulp capping with the application of a 3-step total-etch adhesive system (OptiBond FL, Kerr, Brea, CA, USA). A gel etching agent (Kerr Etchant Gel, Krea, CA) with 37.5% phosphoric acid was placed on the enamel and dentin for 15 s and then rinsed with water until the etchant was completely removed (approximately 15 s); subsequently, it was gently air-dried for a few seconds, being careful not to desiccate dentin. Afterwards, OptiBond FL Primer (Bottle #1) was applied with a micro-brush over enamel and dentin surfaces with a light scrubbing motion for 15 s and then gently air-dried for approximately 5 s. Finally, OptiBond FL Adhesive (Bottle #2) was applied over the enamel and dentin uniformly, creating a thin coating. A light application of air was used to blow the adhesive to the margin or to thin it, and a light-curing for 20 s with an LED curing light at 1000 mW/cm^2^ was performed (Startlight, Mectron, Carasco, Italy). 

Group 5: Indirect pulp capping with the application of 2-step self-etch adhesive system (Clearfil SE Bond, Kuraray, Tokyo, Japan). The primer was applied with a micro-brush on the cavity walls, left in place for 20 s and, after conditioning the tooth surface, the volatile ingredients were gently evaporated with an air stream. Next, the bonding agent was applied with a micro-brush to the entire surface of the cavity, and a gentle oil-free air stream was used to make the bond film as uniform as possible. Additional application of the bond and a subsequent gentle air stream were made, and finally, light-curing for 20 s with an LED curing light at 1000 mW/cm^2^ was performed (Starlight, Mectron, Carasco, Italy). 

The adhesive system employed for Groups 1, 2, 3, and 5 was a 2-step self-etch (Clearfil SE Bond, Kuraray, Tokyo, Japan), while in Group 4 a 3-step total-etch adhesive system (OptiBond FL, Kerr, Brea, CA, USA) was used. Afterwards, the direct restorations for each group were completed with a nano-hybrid composite (Filtek Supreme XTE, 3M ESPE, St. Paul, MN, USA), layered with an oblique technique. Each layer was 2 mm thick and light-cured for 20 s with an LED light at 1000 mW/cm^2^ (Starlight, Mectron, Italy). Final finishing and polishing were performed during the same appointment, using a fine-grit needle-shaped contouring diamond bur (#8858314014, Komet Dental, Lemgo, Germany), rubber points (Supra, Heraeus Kulzer, Hanau, Germany), and flexible discs and finishing strips (Sof-Lex, 3M ESPE, St. Paul, MN, USA). 

### 2.4. Clinical and Radiographic Evaluation 

For the retrospective analysis, the follow-up clinical and radiographical data were collected between January and July 2023. The data considered for the study were thermal and electric pulp vitality tests, percussion tests, and periapical radiographs performed by two calibrated operators who did not participate in the clinical procedures and who blindly evaluated patients. The thermal pulp tests were performed by applying an ice-cold cotton pellet at the cervical tooth crown portion. Teeth that maintained pulp vitality and did not show any clinical or radiographic signs and/or symptoms of irreversible pulpitis or pulp necrosis were considered successes. Otherwise, the teeth with no response to the pulp vitality test and/or with clinical or radiographic signs and/or symptoms of irreversible pulpitis or pulp necrosis were considered failures. The teeth affected by extensive secondary carious lesions were excluded from the analysis.

### 2.5. Statistical Analyses

For the direct pulp-capping groups, a chi-square test was used to study the success rate (significance level was set at *p* < 0.05) and to investigate the association between the event (pulp necrosis) and the pulp-capping material employed (Group 1, Group 2, and Group 3). Intra-operative factors (bleeding level of exposed pulp and width of exposure area) and patient age were also analyzed in relation to successes and failures using a chi-square test (significance level was set at *p* < 0.05). For the indirect pulp-capping teeth groups, a second chi-square test was used to study the success rate (significance level was set at *p* < 0.05) and to investigate the association between the event (pulp necrosis) and the adhesive system employed (Group 4 and Group 5). Direct and indirect pulp-capping successes were analyzed with the parameters evaluated during recalls (symptoms, thermic test, electric test, percussion test, and presence of pulp calcifications in periapical radiography) and using a chi-square test to investigate the association between recall parameters and the type of capping procedure.

## 3. Results 

In this retrospective clinical study, 140 patients (84 females and 56 males) were evaluated. At the initial visit, when the direct/indirect pulp-capping procedures were performed, there were 12 patients under the age of 18, 20 patients between 19 and 25, 32 aged 26–40, 60 aged 41–60, and 16 over 60 years old. Dental-capping procedures were performed to treat 276 permanent teeth: 180 maxillary and 96 mandibular, with 24 incisors, 116 premolars, and 136 molars. Among them, 128 teeth were treated from female patients (46.4%) and 148 from male patients (53.6%). Furthermore, 48 treatments were performed on patients under 18 years old, 28 on patients aged 19–25 years, 80 aged 26–40, 88 aged 41–60, and 32 on patients over 60 years old (Table 1). All patients were recalled after 10 years: the follow-up time varied from 96 months to 151 months, with a mean observation period of 126.32 months (±17.02), and the overall success rate was 72.5% (200 successes, 76 failures).

### 3.1. Direct Pulp Capping

Of all the permanent teeth included in the study, 84 were treated with direct pulp capping: 32 (38.1%) with Mineral Trioxide Aggregate cement (Group 1), 32 (38.1%) with a resin-based glass ionomer (Group 2), and 20 (23.8%) with TheraCal (Group 3). Sixty (71.4%) treatments were completed on females and twenty-four (28.6%) on males. A total of 12 treatments were carried out on patients under 18 years old, 8 on patients aged 19–25 years, 16 aged 26–40, 32 aged 41–60, and 16 on patients over 60 years old. The mean follow-up was 106.67 months (±8.54) for direct pulp-capping treatments. The success rate in direct pulp capping was 23.8%.

In teeth treated with direct pulp capping, the success rate was 37.5% in Group 1, 0% in Group 2, and 40% in Group 3. Notably, the direct pulp-capping treatments showed a statistically significant association between pulp necrosis and the pulp-capping material according to the chi-square test used to examine the relationship between the event (pulp necrosis) and the pulp-capping material used (*p* = 0.017). The chi-square tests also revealed a correlation between the event (pulp necrosis) and the width of the pulp exposure area before direct pulp capping (*p* = 0.035), but not with bleeding (*p* = 0.053) (Table 2). Another factor that showed a correlation with pulp vitality maintenance was patient age (*p* = 0.013), with a significantly increased amount of pulp necrosis occurring in patient over 25 years old (Table 2). Interestingly, a sub-group analysis that excluded Group 2 reinforced the correlation between the pulp vitality maintenance and the exposure area (*p* = 0.029) and patient age (*p* = 0.01) in Groups 1 and 3. Nevertheless, this analysis reported a correlation between the event and the bleeding (*p* = 0.05). Pulp calcifications were discovered in 80% of direct capping recalls through periapical radiographs. The correlation between the formation of pulp calcifications and the type of capping procedure was confirmed by a chi-square test (*p* = 0.001).

### 3.2. Indirect Pulp Capping

A total of 192 permanent teeth were treated with indirect pulp capping: 88 (45.8%) with the three-step total-etch adhesive system (Group 4) and 104 (54.2%) with the two-step self-etch adhesive system (Group 5). A total of 68 (35.4%) treatments were completed on females and 124 (64.6%) on males. A total of 36 treatments were carried out on patients under 18 years old, 20 on patients aged 19–25 years, 64 aged 26–40, 56 aged 41–60, and 16 in patients over 60 years old. The mean follow-up was 134.92 months (±11.88) for indirect pulp-capping treatments. The success rate increased to 93.8% in indirect pulp capping. In particular, in teeth treated with indirect pulp capping, the success rate was 100% in Group 4 and 88.5% in Group 5. The chi-square used to examine the relationship between the event (pulp necrosis) and the adhesive system employed in the indirect pulp-capping treatments showed that there was a significant relationship between them (*p* = 0.019). The statistical analysis also revealed a correlation between pulp necrosis and patient’s age (*p* = 0.021), with a significantly higher number of failures in patients over 40 years old (Table 2). The pulp calcifications were discovered in 33% of indirect capping recalls through periapical radiographs. Finally, the type of tooth was not considered related to the indirect pulp-capping outcomes (*p* = 0.12).

## 4. Discussion

This study evaluated the long-term prognosis of direct and indirect pulp-capping treatments finalized by direct composite restorations. Based on the results, a possible correlation between patient age and the materials used for the direct and indirect pulp-capping treatments and the preservation of pulp vitality over time was suggested. 

In this study, the overall success rate of the direct pulp-capping procedures is lower than other reports [23]. However, the long-term follow-up, the selected materials, and the pulp diagnosis could have influenced this aspect [20]. When performing a direct pulp-capping treatment, it is important to consider the diagnosis, the clinical procedure, and the material selection [24]. 

The data of the pulp diagnosis recorded through thermal and electric pulp tests were considered. The inclusion criteria stated the absence of pulp symptoms and spontaneous or percussion pain. This indicated to exclude the condition of irreversible pulpitis, which could require coronal pulpotomy or pulpectomy [25]. Therefore, according to Wolters et al., the clinical situations of moderate and severe pulpitis were excluded from the pulp-capping treatments [25]. However, a recent review reported that scientific evidence to clinically determine the true pulp status is lacking [26]. Nevertheless, spontaneous pain was indicated as one of the most promising indicators for irreversible pulp inflammation, and this was considered among the exclusion criteria [26]. 

During the clinical follow-up, the electric pulp test values were considered variable depending on the type of tooth, the patient age, and subjectivity [27]. Therefore, the electric pulp test values were considered positive (vital pulp) or negative (pulp necrosis) in order to discriminate between these two different clinical situations. Similarly, the presence of percussion pain was observed in necrotic teeth, being this dichotomous value related to the absence of pulp vitality. 

The exposed pulp was not subjected to disinfection or hemostasis using oxygen peroxide or sodium hypochlorite (NaOCl). Even if NaOCl use is considered safe and it has been recently suggested [28], it has also been indicated to have a possible toxic effect on pulp tissue and dentin, reducing the bond strength [29,30]. Although some other solutions have been proposed to stop pulp bleeding, they may be cytotoxic, influencing the study outcomes [21,30]. Therefore, when minimal pulp bleeding was present, pulp-capping materials were applied before a 1 min compression with a physiologic solution [31,32,33]. 

In the current trial, the resin-based glass ionomer exhibited a lower probability of maintaining pulp exposure vitality compared with other materials, supporting some previous findings [34,35,36]. Even if fluoride has been claimed to be beneficial due to the formation of fluoride–apatite crystals and remineralization [10,37], the diffusion of glass ionomer components into the pulp may result in long-lasting inflammation mediated by macrophages and giant cells that impairs healing and dentinal bridge formation [34,35,36,37]. 

Therefore, the most effective material for direct pulp capping is thought to be the MTA. The MTA and calcium hydroxide have similar mechanisms of action: when in contact with pulp, the calcium hydroxide produced by hydrating MTA is leached out, resulting in tissue necrosis [38]. However, the benefits of the MTA are thought to be its ability to seal, its high biocompatibility, its bioactivity, and its capacity to encourage the formation of mineralized tissue [18,39,40]. Additionally, the MTA is claimed to be superior to calcium hydroxide due to dentin bridge formation that is more uniform and thicker, as well as less inflammatory reaction and pulp necrosis [18,39,40]. Therefore, our results are in accordance with the literature, where the MTA has been studied as a material for direct pulp capping, demonstrating less inflammation, less hyperemia, odontoblast-like cell differentiation, and the formation of a thicker dentinal bridge [24,32,33,34,35,36,37,38,39,40,41,42]. Despite its many benefits, the MTA has some drawbacks, such as prolonged setting times, poor handling, and coronal tooth discoloration [43]. 

On the other hand, when compared to the MTA or Biodentine, TheraCal demonstrated slightly lower pulp vitality maintenance outcomes and worse histologic findings [44]. These results may be explained by the low MTA concentration and the presence of resin in the TheraCal composition [44]. However, previous studies reported that TheraCal releases a higher calcium rate than the MTA and calcium hydroxide [6,45,46], and Ca release enables odontoblast differentiation and the expression of proteins related to calcification [6,44]. Nevertheless, the incomplete hydration, the lower calcium hydroxide formation, and the impossibility to obtain a complete polymerization of TheraCal justify the possible formation of incomplete dentinal bridges after the apposition of this material [11,38,44,45,46,47].

The chi-square tests regarding intra-operative factors revealed a correlation between pulp necrosis and the width of the exposure area before direct pulp capping (*p* = 0.035), but not with bleeding (*p* = 0.053). Noteworthy, direct pulp-capping failures may be attributed to the unstable clinical circumstances present during the procedures, such as bleeding and delayed hemostasis. Therefore, for direct pulp capping to be successful, bleeding should be perfectly controlled, and blood clots should not be present between the capping material and the pulp tissue. Clinically, it is difficult to apply a capping material to a wet, bloody surface, and the presence of blood has been associated with a higher risk of post-operative infection [47]. However, this study failed to display a correlation between the presence of bleeding and the occurrence of pulp necrosis during follow-up. This result seems in accordance with a previous review that indicated that the amount and time of pulpal bleeding after exposure are not related to treatment outcomes [29]. Nevertheless, direct pulp-capping failures could happen more frequently in the case of bleeding, probably due to the presence of several bioactive molecules within blood clots [48]. Thus, a sub-group analysis of only Groups 1 and 3 revealed a slight correlation between pulp necrosis and the occurrence of bleeding (*p* = 0.05), probably due to the exclusion of the confusing action of the material used in Group 2.

Over time, patient age also affected the results of direct pulp capping (*p* = 0.013). Younger patients present better treatment outcomes, according to some previous studies [49]. Some other studies were unable to support the effect of age on direct pulp-capping outcomes [49], even if the different age groups examined may be the cause of this discrepancy. In the current study, 87.5% of failures were reported in patients over 25 years old, with a significantly higher incidence of pulp necrosis. This result is consistent with the noticeably lower survival rate for patients over 40 that has been reported in other studies [25,49,50]. The migration, proliferation, and mineralization of the pulp cells orchestrates pulp wound healing, which includes the formation of reparative dentin. However, when pulp is exposed and the pre-existing odontoblastic layer is damaged, reparative dentin is created by odontoblast-like cells that are likely differentiated from DPSCs (dental pulp stem cells) [3]. The number of DPSCs and their differentiation mechanisms are diminished in older patients, with a subsequent lower ability to produce reparative dentin. Therefore, an age of 25 years or less is a protective factor from pulp necrosis occurrence after direct pulp-capping procedures [51]. 

Regarding the indirect pulp-capping treatments, the success rate was 100% for the teeth treated with the total-etch adhesive system and 88.5% for the teeth treated with the self-etch adhesive system. This indicated a significant correlation between pulp vitality preservation and the adhesive system when indirect pulp-capping treatments were considered. Thus, the secondary null hypothesis was rejected. Usually, self-etch dentin adhesives are thought to reduce post-operative hypersensitivity to a greater extent than etch-and-rinse systems due to their ability to expose fewer dentinal tubules, maintaining the residual smear plugs [19,21]. However, the systems’ lower acidity toward the dental substrate made them less effective regarding their sealing ability [42,43,44,45,46,47,48,49,50,51,52,53]. Notably, in the current retrospective in vivo study, the etch-and-rinse technique did not demonstrate a dramatic failure of the indirect pulp-capping procedures. Therefore, it could be hypothesized that the application of phosphoric acid over enamel and dentin along with the use of a multistep adhesive system can produce a stable and long-lasting hybrid layer [19,20]. This approach could be beneficial for the maintenance of the pulp integrity over time. However, to confirm the present findings, a larger population is required with the introduction of different materials indicated for the pulp-capping procedures.

## 5. Conclusions

This retrospective study showed that the pulp-capping material should be carefully considered when performing a direct capping procedure. It also demonstrated that vitality preservation depends on patient age and the width of the exposed pulp tissue area. Similarly, the effectiveness of vitality preservation in indirect pulp-capping treatments depends on the adhesive system, with the total-etch system outperforming the self-etch system.

## Figures and Tables

**Table 1:** The cohort characteristics at baseline.

Treated Teeth (n = 276)		Total (%)
Patient age (years)	<18	17.4
	19–25	10.2
	26–40	28.9
	41–60	31.9
	>60	11.6
Teeth	incisors	8.7
	premolars	42.1
	molars	49.2
Patient gender	male	53.6
	female	46.4

**Table 2:** Correlation between pulp necrosis and the material used for direct and indirect pulp capping procedures, the width of the pulp exposure, and the bleeding before direct capping and patient age (*p* < 0.05).

Variables	Values	Success (%)	*p*-Value
Direct pulp-capping material	MTA	37.5	0.017
	Resin-based glass ionomer	0.0	
	TheraCal	40.0	
Indirect pulp-capping material	3-Step total-etch adhesive	100.0	0.019
	2-Step self-etch adhesive	88.5	
Width of the pulp exposure area before direct pulp capping	<1 mm	100.0	0.035
	>1 mm	66.7	
Bleeding before direct pulp capping	Absent	80.0	0.053
	Minimal	20.0	
	Moderate	0.0	
Age (years)	<18	40.0	0.013
	19–25	20.0	
	26–40	12.5	
	41–60	0.0	
	>60	0.0	

## Data Availability

The data presented in this study are available on request from the corresponding author due to hospital privacy policy.
